# Myoanatomy and serotonergic nervous system of the ctenostome *Hislopia malayensis*: evolutionary trends in bodyplan patterning of ectoprocta

**DOI:** 10.1186/1742-9994-8-11

**Published:** 2011-05-16

**Authors:** Thomas Schwaha, Timothy S Wood, Andreas Wanninger

**Affiliations:** 1 University of Vienna, Department of Morphology, Althanstraße 14, 1090 Vienna, Austria; 2 Wright State University, Department of Biological Sciences, 3640 Colonel Glenn Highway Dayton, OH 45435 USA

## Abstract

**Background:**

Ectoprocta is a large lophotrochozoan clade of colonial suspension feeders comprising over 5.000 extant species. Their phylogenetic position within the Lophotrochzoa remains controversially discussed, but also the internal relationships of the major ectoproct subclades -Phylactolaemata, Stenolaemata, and Gymnolaemata - remains elusive. To gain more insight into the basic configuration of ectoproct muscle systems for phylogenetic considerations, we analysed the adult myoanatomy and the serotonergic nervous system as well as myogenesis in budding stages of the ctenostome *Hislopia malayensis*.

**Results:**

In adults, the serotonergic nervous system is restricted to the lophophoral base with a high concentration in the cerebral ganglion and serotonergic perikarya between each pair of tentacles. Prominent smooth apertural muscles extend from the basal cystid wall to each lateral side of the vestibular wall. The musculature of the tentacle sheath consists of regular strands of smooth longitudinal muscles. Each tentacle is supplied with two bands of longitudinal muscles that show irregular striation. At the lophophoral base several muscles are present: (i) Short muscle fibres that proximally diverge from a single point from where they split distally into two separate strands. (ii) Proximally of the first group are smooth, longitudinal fibres that extend to the proximal-most side of the lophophoral base. (iii) Smooth muscle fibres, the buccal dilatators, traverse obliquely towards the pharynx, and (iv) a circular ring of smooth muscle fibres situated distally of the buccal dilatators. Retractor muscles are mainly smooth with short distal striated parts. The foregut consists mainly of striated ring musculature with only few longitudinal muscle fibres in the esophagus, while the remaining parts of the digestive tract solely exhibit smooth musculature. During budding, apertural and retractor muscles are first to appear, while the parietal muscles appear at a later stage.

**Conclusions:**

The apertural muscles show high similarity within Ectoprocta and always consist of two sets of muscles. Gymnolaemates and Phylactolaemates show clear differences within their digestive tract musculature, the former showing smooth and longitudinal muscles to a much greater extent than the latter. The complex musculature at the lophophoral base appears promising for inferring phylogenetic relationships, but sufficient comparative data are currently lacking.

## Introduction

The lophotrochozoan phylum Ectoprocta consists of benthic, colonial filter feeders that live on various substrates. It currently contains over 5.000 extant and approximately 20.000 described extinct species. They are currently assigned to three taxa, whereby the Phylactolaemata represent a small group of solely freshwater-inhabiting ectoprocts, while the Stenolaemata (with the only remaining extant taxon Cyclostomata) and the Gymnolaemata mainly constitute marine animals. Within the Gymnolaemata two distinct groups, the Ctenostomata and Cheilostomata, are recognized [1]. The relationships between these higher taxa are currently not well understood: The Phylactolaemata are considered monophyletic, while the Cheilostomata and Stenolaemata have been considered both monophyletic [2] or polyphyletic [3,4]. Due to their calcified protective skeletons, the latter two taxa show a long fossil record yielding more insight into their evolution. In contrast, the fossil record of the non-mineralized Ctenostomata is poor [5] and only represented by casts of borings (e.g. [6]) and bioimmurations (i.e., overgrowth by encrusting, mineralized organisms, cf. [7]).

There is a broad consensus that "Ctenostomata" is paraphyletic and that ctenostome-like ancestors have led independently to the origin of the calcified exoskeletons of Cheilostomata and Stenolaemata [4,5,8,9]. Accordingly, the ctenostomes are of particular interest for studies dealing with ectoproct evolution. Due to the scarcity of their fossil record, the study of extant ctenostome ectoprocts appears particularly promising for further insights into ectoproct origins and phylogenetic relationships. To date, ctenostome phylogenies are mostly based on features of the cystid and colony morphology [5,10], while details on the anatomy of the soft body (polypide) remain less investigated and are thus widely neglected in phylogenetic analyses.

Myoanatomical features and neurotransmitter distribution, for example serotonin or FMRF-amide, have recently been used for phylogenetic inferences among lophotrochozoans [11-13]. Thereby, several immunocytochemical investigations have dealt with the neuromuscular system of different ectoproct larval types [14-21], whereas no such study is as of yet available for adult ectoprocts. Data on the development of the zooids and the ontogenetic appearance of certain muscle systems have been used to elucidate ectoproct internal relationships [3,22-25]. Myoanatomical details of the digestive tract have also been proposed to be useful to discriminate certain subtaxa [26,27]. By contrast, characters of the nervous system have never been considered on a broad, comparative scale for systematic or phylogenetic deductions.

**Figure 1:**
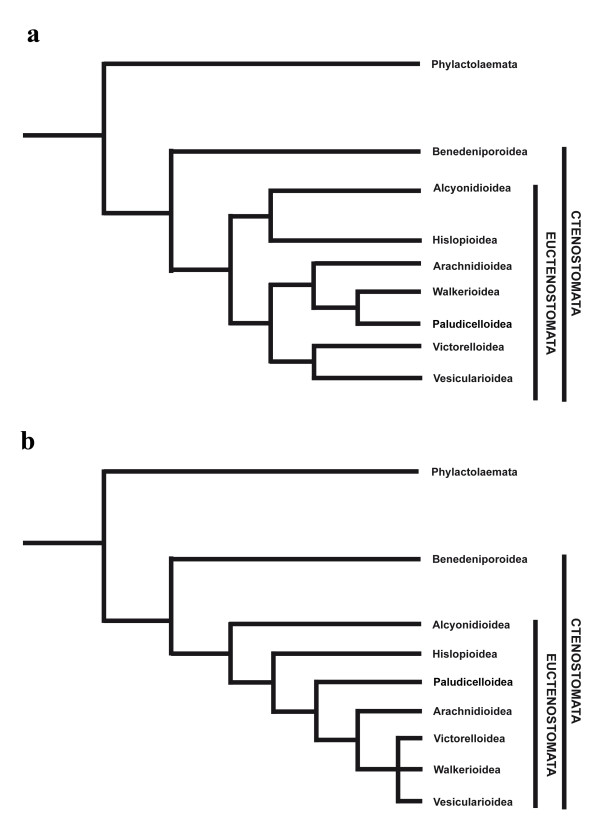
**Phylogenetic system of the Ctenostomata, modified after (a) Jebram (1986) and (b) Todd (2000)**. (a) The phylogenetic reconstruction of Jebram is mainly based on cystid and muscle differentiation. (b) The work of Todd (b) has in particular included characters of fossil ctenostomes.

Hislopiid ctenostomes comprise only seven freshwater species and are the sole family within the superfamily Hislopioidea. The latter belongs to the paraphyletic 'carnosans', which are regarded as primitive within the Euctenostomata (sensu [25]), and which have retained a simple colonial morphology similar to the proposed cheilostome-like ancestor (see e.g. [5,9,22,25,28]). Their supposedly relatively basal position within Euctenostomata (Figure 1; [5,10,22,25]) renders the Hislopiidae an important model taxon for inferring ectoproct phylogeny and evolution. To gain more insight into the basic configuration of ectoproct muscle systems and to evaluate their potential use for phylogenetic studies, we analyzed the adult myoanatomy and serotonergic nervous system as well as myogenesis during budding in *Hislopia malayensis *Annandale, 1916.

## Materials and methods

### Animals

Colonies of *Hislopia malayensis *Annandale, 1916 were collected from the pond of the Faculty of Fisheries of the Kasetsart University in Bangkok (see [29]). Specimens were fixed in 4% paraformaldehyde in 0.01 M phosphate buffer (PBS) containing 0.01% NaN_3 _for 1 hour at room temperature. Subsequently, they were rinsed three times for 20 min and stored in the same solution.

### Immunocytochemistry and confocal microscopy

Some colonies were dissected prior to staining to increase tissue permeability. For F-actin staining, specimens were permeabilized in PBS containing 4% Triton-X (PBT) for 1 hour, followed by overnight incubation in a 1:40 dilution of AlexaFluor 488 phalloidin (Molecular Probes, Eugene, OR, USA) in PBT at 4°C. Then, the specimens were rinsed three times in PBS. For staining of the serotonergic nervous system, pieces of *H. malayensis *colonies were transferred to 6% normal goat serum (NGS; Sigma-Aldrich, St. Louis, MO, USA) in PBT (block-PBT) overnight at 4°C. Subsequently, a polyclonal rabbit anti-serotonin antibody (Zymed, San Francisco, CA, USA) was applied at a concentration of 1:400 in block-PBT for 24 hours at 4°C. Then, the specimens were rinsed several times in block-PBT for 6 hours at 4°C prior to application of a secondary fluorochrome-conjugated antibody (goat anti-rabbit AlexaFluor 594, Molecular Probes) in block-PBT at a concentration of 1:200 for 24 hours at 4°C. Specimens were then washed three to four times in PBS for about 6 hours. Negative controls were performed by omitting the primary antibody and yielded no signal. Nuclei were stained by adding a few drops of DAPI (Invotrogen, 3 μg/ml) for 15-20 minutes, followed by three short washes in PBS. Specimens were mounted in Fluoromount G (Southern Biotech, Birmingham, AL, USA) on standard microscope slides.

Analysis and image acquisition was performed on a Leica DM IRBE microscope equipped with a Leica TCS SP2 confocal unit (Leica Microsystems, Wetzlar, Germany). Confocal image stacks were recorded with 0.5-1μm step size along the Z-axis. Images stacks were captured as maximum intensity projections or further processed as volume renderings with Amira 4.1 software (Mercury Computer Systems, Chelmsford, MA, USA).

## Results

### Myoanatomy of adult *Hislopia malayensis*

**Figure 2:**
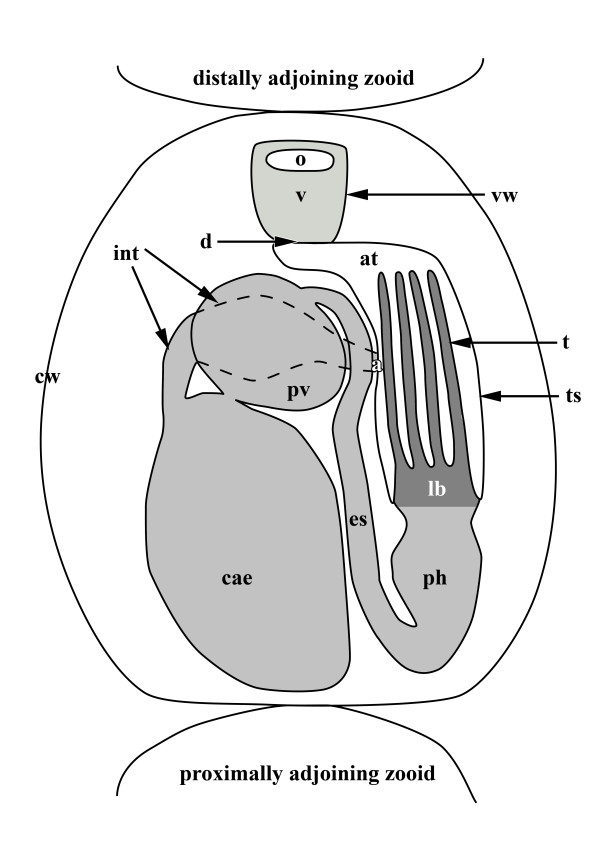
**Schematic overview of a retracted *Hislopia malayensis *zooid showing the main components of the polypide**. The orifice at the distal end of the zooid reaches into the vesitbulum, which is separated from the proximally situated tentacle sheath by the diaphragm. Within the tentacle sheath the tentacle crown (i.e. the lophophore) is situated. The mouth opening is situated at the lophoral base and leads into the broad pharynx which is followed by an elongated, tube-like esophagus. The latter continues into the prominent proventriculus from where the digestive tract leads into the voluminous caecum. From the caecum the intestine leads into the anus, which terminates in the tentacle sheath.Abbreviations: a - anus, at - atrium, cae - caecum, cw - cystid wall, d - diaphragm, es - esophagus, int - intestine, lb - lophophore base, o - orifice, pv - proventriculus, t - tentacle, ts - tentacle sheath, v - vestibulum, vw - vestibular wall.

**Figure 3:**
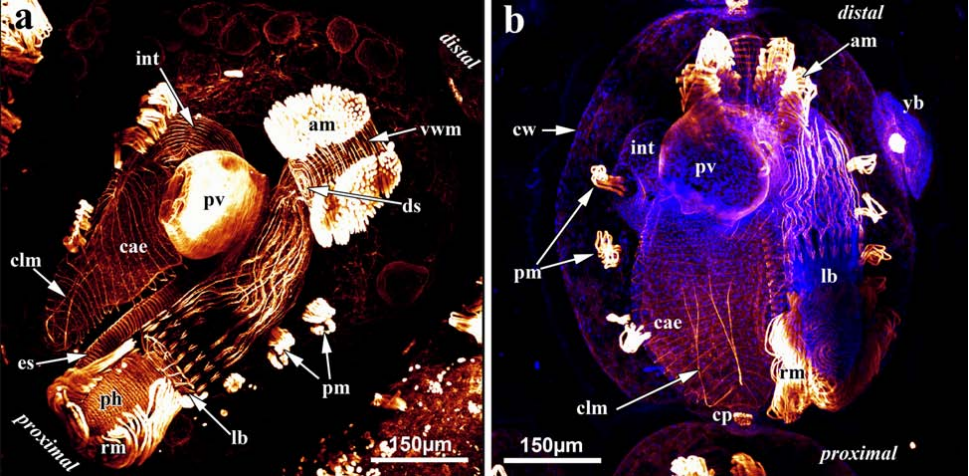
**Maximum-intensity projections of confocal laserscanning image stacks providing an overview of the muscle system of a single zooid of *Hislopia malayensis***. (a) F-actin staining. Solely the parietal muscles are associated with the cystid. Most muscles of the zooid are present at the distally situated aperture, which continues proximally into the polypide. Prominent retractor muscles run from the distal end of the zooid to the lophophoral base. From the lophophoral base the digestive tract starts with the pharynx, followed by the esophagus, which both possess mostly striated ring musculature. The adjoining proventriculus is the most prominent region of the digestive tract and possesses only smooth ring musculature. The caecum carries several distinct ring muscles and two longitudinal muscles at its proximal tip. The intestine possesses only smooth, longitudinal musculature. (b) Different zooid of *H. malayensis *similar as in (a) but also with cell nuclei stained with DAPI to provide a clearer picture of the cystid wall and its outlines. Also note the early bud on the right side. Abbreviations: am - apertural muscles, cae - caecum, clm - caecal longitudinal muscle, cp - communication pore, cw - cystid wall, ds - diaphragmatic sphincter, es - esophagus (cardia), int - intestine, lb - lophophore base, o - orifice, pm - parietal muscles, pv - proventriculus, rm - retractor muscles, ts - tentacle sheath, v - vestibulum, vwm - vestibular wall musculature, yb - young bud.

**Figure 4:**
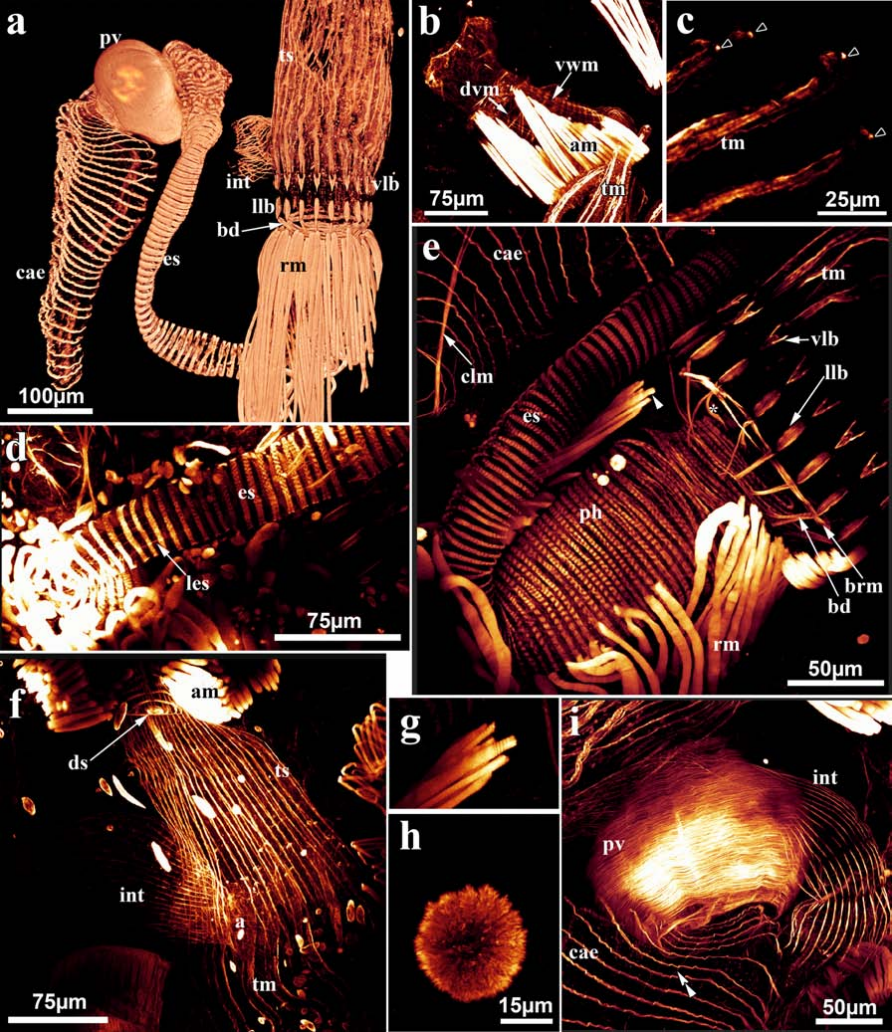
**Myoanatomical details of the polypide of *Hislopia malayensis***. All but (a) are maximum-intensity projections of confocal laserscanning image stacks. (a) Volume rendering of the confocal stack of the myoanatomy of a dissected polypide showing most of the musculature of the digestive tract. (b) Oblique view on the vestibular wall showing apertural muscles and vestibular wall muscles. Diagonal vestibular wall muscles can be distinguished on the basal side. (c) Detail of the tentacle musculature and muscular concentration at the tentacles tips (open arrowheads). (d) Musculature of the esophagus showing thin longitudinal muscle fibres. (e) Lophophoral base musculature and parts of the digestive tract. Asterisk marks a second pair of buccal dilatators. (f) Detail of the tentacle sheath musculature and apertural muscles. (g) Magnified view of the distal portion of the retractor muscles displayed in (e). (h) Muscular elements of a rosette plate between individual zooids. (i) Musculature of the proventriculus and adjacent parts of the digestive tract. Double arrowheads marks fine longitudinal muscle fibres in the caecum. Abbreviations: a - anal area, am - apertural muscles, bd - buccal dilatators, brm - ring muscle at the lophophore base, cae - caecum, clm - caecal longitudinal muscle, ds - diaphragmatic sphincter, dvm - diagonal vestibular wall musculature, es - esophagus, int - intestine, lb - lophophore base, les - longitudinal musculature of the esophagus, llb - longitdunal muscles at the lophophore base, pm - parietal muscles, pv - proventriculus, rm - retractor muscles, tm - longitudinal tentacle muscles, ts - tentacle sheath, vlb - v-shaped muscles at the lophophore base, vwm - vestibular wall musculature, yb - young bud.

Colonies of *Hislopia malayensis *form simple encrusting sheets on a variety of artificial or natural substrates [30]. Its individual zooids are flat and oval-shaped and are interconnected by rosette-shaped communication pores. Each zooid consists of a more or less rigid, chitinous to hyaline protective cystid and a flexible polypide that contains all major organs of the zooid (Figure 2). On each side of the zooid a series of four to five parietal muscle bundles are associated with the cystid wall. They originate from the basal attachment site and traverse the coelomic cavity to the frontal cystid wall, where the orifice is situated (Figure 3a, b). The inner plug of each communication pore shows a high number of actin-filaments, which is highest in its periphery and decreases towards the center (Figure 4h).

In retracted zooids the polypide is attached to the cystid by an almost rectangularly invaginated vestibulum that extends from the orifice on the frontal cystid wall to the diaphragm at the distalmost end of the invaginated tentacle sheath (Figure 2). Prominent apertural muscles extend from the basal cystid wall to each lateral side of the vestibular wall. They consist of smooth muscle fibres and extend along the entire length of the vestibular wall (Figure 3a, b; 4b). On the frontal and lateral side, the vestibular wall shows a regular net of smooth ring and longitudinal musculature, whereas diagonal and longitudinal muscles are present on the basal side (Figure 4b). A sphincter at the diaphragm (diaphragmatic or atrial sphincter) separates the vestibulum from the space enclosed by the tentacle sheath, the atrium (Figure 2;3a;4f). The tentacle sheath stretches from the diaphragm to the lophophoral base. Its musculature consists of regular strands of smooth longitudinal muscles that run from its distalmost end approximately to the region of the anal area (Figure 4f). At its proximal end, the tentacle sheath continues into the lophophore. Each tentacle is supplied with two bands of longitudinal muscles that show irregular striation (Figure 4b, c, e). A distinct concentration of actin is present at the distal tip of each tentacle (Figure 4c). Proximally, the longitudinal tentacle musculature extends to the lophophoral base. At the lophophoral base four groups of muscles are present: Below the musculature of each tentacle, short muscle fibres are found that proximally diverge from a single point from where they split distally into two separate strands. These muscle elements are approximately V-shaped with some fibres traversing medially (Figure 4a, e). A second set of muscles is situated proximally to the first group and consists of smooth, longitudinal fibres that extend to the proximal-most side of the lophophoral base (Figure 4a, e). From that point smooth muscle fibres, the buccal dilatators, traverse obliquely towards the pharynx (Figure 4a, e). At the site of the cerebral ganglion, two pairs of buccal dilatators are present. The first pair inserts more proximally on the pharynx, whereas the second pair attaches more distally to the pharynx (Figure 4e). A circular ring of smooth muscle fibres is situated in the region of the mouth, medially to the proximal-most part of the lophophoral base, and slightly above the pharynx (Figure 4e). The retractor muscles of the polypide originate from the proximal cystid wall and insert at the entire lophophoral base except for the ganglion area (Figure 3a; Figure 4a, e). The fibres of the retractor muscles appear smooth for most of their length. In some cases, however, the distal-most part of the fibres appears cross-striated (Figure 4g). The pharynx below the mouth opening is provided with several narrow bands of circular musculature which shows distinct cross-striation (Figure 3a;4e). The pharynx continues into the esophagus, which is an elongated tube. Similar to the pharynx, its musculature is mainly composed of obliquely striated ring musculature (Figure 2;3a;4a, e), but few delicate longitudinal muscle fibres could be observed as well (Figure 4d). The following cardia or proventriculus is bulbous and possesses densely packed smooth ring musculature (Figure 3a;4a, i). The digestive tract continues into the voluminous caecum which for the most part carries bands of smooth ring musculature (Figure 3a, b;4a, e, i). From the proximal end of the caecum two prominent longitudinal muscles extend distally. Halfway of the stomach they split into two bundles (Figure 3a, b;4e). A few sparse longitudinal muscle fibres are also present between the circular muscle bands (Figure 4i). The intestine, which adjoins the stomach, carries smooth longitudinal muscles over its entire length and terminates in the tentacle sheath (Figure 3a;4a, f, i).

### Myogenesis in budding zooids

**Figure 5:**
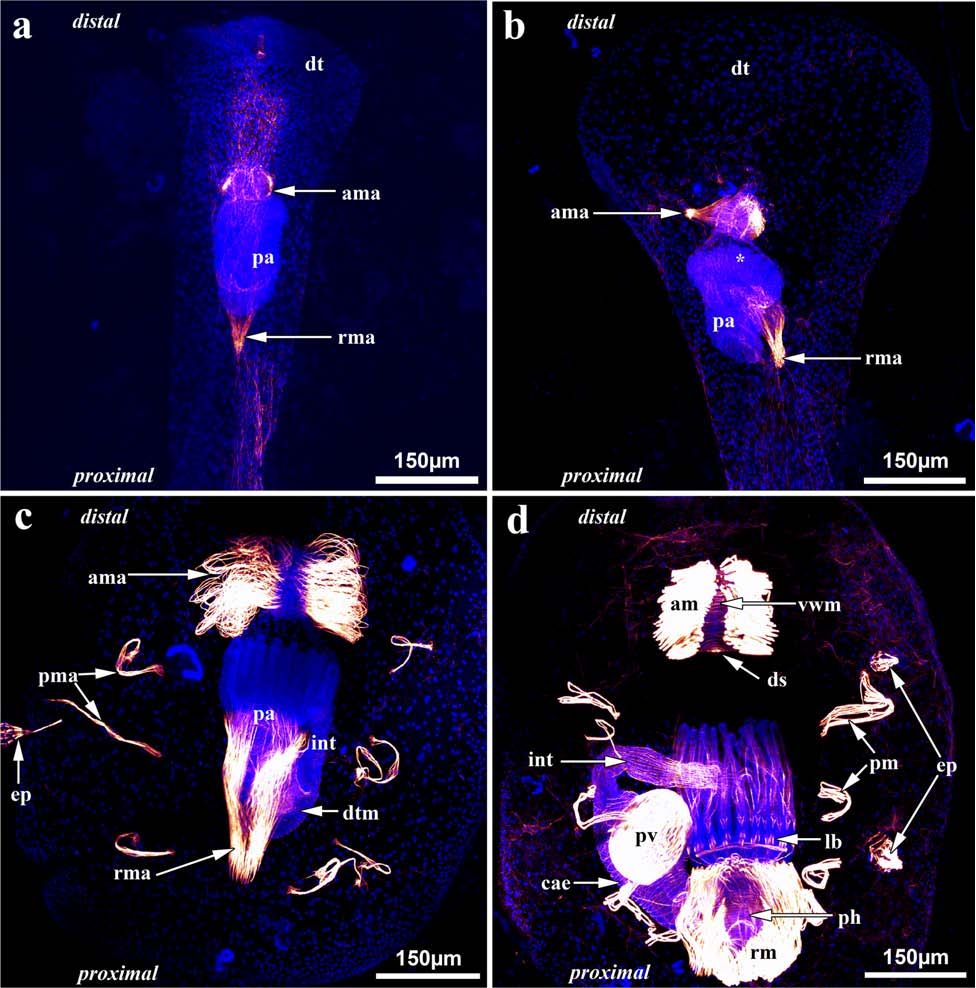
**Myogenesis during the budding process of *Hislopia malayensis***. Maximum-intensity projections of confocal laserscanning image stacks. (a) Early budding stage showing the polypide anlage and the first anlagen of the developing apertural and retractor muscles at its distal and proximal side, respectively. (b) More advanced budding stage with distinct tentacle anlagen (asterisk), in which the cystid of the bud has widened distally and the apertural and retractor muscle anlagen have grown and are more pronounced. (c) Advanced budding stage where first anlagen of the digestive tract musculature have formed and the parietal muscles are present laterally of the polypide anlage. Both, the apertural and the retractor muscle anlagen are most prominent. The former consists of loosely smooth muscle fibres, whereas the prospective retractor muscles have differentiated into two elongated muscle fibre bundles. (d) Almost completely developed zooid where most parts of the digestive tract, especially the most prominent proventriculus, are formed similar to the adult. Compare to Figures 3 and 4 for details on the musculature of adult specimens. Abbreviations: am - apertural muscles, ama - apertural muscle anlage, cae - caecum, ds - diaphragmatic sphincter, dt - distal tip of the cystid of the bud, dtm - developing digestive tract musculature, ep - epizooic organism, int - intestine, lb - lophophore base, pa - polypide anlage, pma - parietal muscle anlage, pm - parietal muscles, pv - proventriculus, rm - retractor muscles, rma - retractor muscle anlage, vwm - vestibular wall musculature.

Buds start as distal or lateral outgrowths of the cystid of a zooid that soon become constricted off from the mother zooid (Figure 3b). Further development leads to a tube-like elongation of the cystid, slightly broadened at its distal tip. In the middle of the bud the polypide anlage develops. The retractor and apertural muscles are first to appear in the bud. The anlagen of the retractor muscles are V-shaped: Proximally, they originate from the cystid wall from a single site, whereas distally the bundles split and insert at the developing polypide. The apertural muscles form as two small bands on each side of the prospective aperture (Figure 5a).

In a later budding stage with distinct tentacle anlagen, the cystid has broadened distally. The retractor and apertural muscles have only slightly changed, but possess more muscle fibres (Figure 5b). Additional muscles have not formed at this stage of budding. Further in development, the cystid widens even more on its lateral sides. Compared to the previous stage, the widening has also progressed towards the proximal pole of the zooid. Three to four distinct parietal muscle bands appear laterally to the developing polypide. Besides the much enlarged retractor and apertural muscles, musculature of the digestive tract has started to form (Figure 5c). In the oldest stage analysed, the cystid exhibits the oval shape, characteristic of adults. The developing polypide already shows most of the adult musculature. In addition, differentiation and regionalization of the digestive tract has commenced and its corresponding musculature has started to form. The lophophoral base and the tentacles already show all muscular elements that occur in the adults. The apertural muscles are prominent and ring musculature of the duplicature as well as the diaphragmatic sphincter is present. Only the tentacle sheath is still devoid of muscles (Figure 5d).

### Serotonergic nervous system

**Figure 6:**
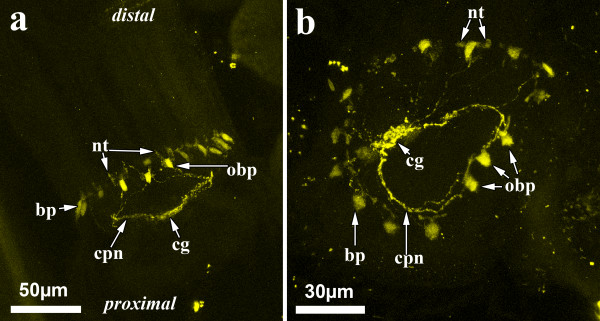
**The adult serotonergic nervous system of *Hislopia malayensis***. Maximum-intensity projections of confocal laserscanning image stacks. (a) Lateral view of the lophophoral base showing the cerebral ganglion at the proximal side and parts of the circumpharyngeal neurite bundles emanating to serotonergic perikarya at the lophophoral base. From the latter, two neurites are distinguishable at the base of each adjacent tentacle. (b) Oral view of the serotonergic nervous system at the lophophoral base. The highest concentration of serotonin is found in the cerebral ganglion on the left side. At the oral side, three serotonergic perikarya are present associated with the circumpharyngeal neurite bundle. Further neurites extend towards the serotonergic perikarya from the ganglion and the circumpharyngeal ring. Abbreviations: cg - cerebral ganglion, cpn - circumpharyngeal neurites, bp - perikarya at the lophophore base, obp - perikarya on the oral side of the lophophoral base, nt - neurites extending into the tentacles.

The serotonergic nervous system in adult zooids is restricted to the lophophoral base of each polypide (Figure 6a, b). The highest concentration is found in the cerebral ganglion, from where neurites extend circumpharyngeally. On the oral side there are three serotonergic perikarya at the base of each pair of tentacles, which are connected to the circumpharyngeal neurites to form a nerve ring. At the remaining tentacles, neuronal perikarya are present as well. These are, however, more apart than the three oral ones and are directly connected to the ganglion via delicate neurites. From each perikaryon short neurites extend into each of the two tentacles.

## Discussion

### Homology of apertural muscles and their phylogenetic significance

**Figure 7:**
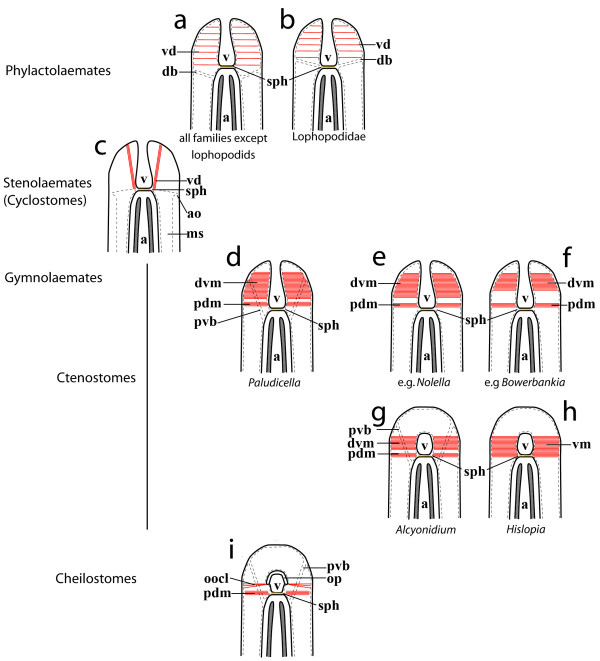
**Schematic representation of apertural areas of retracted ectoproct zooids with associated muscles**. The distal zooidal part points upwards. Epidermal layers are drawn in continuous lines and coelomic epithelia dashed. Tentacles are displayed in dark grey and the diaphragm in orange. (a + b) Apertural muscles in phylactolaemates consist of sparse vestibular dilatators and duplicature bands that either insert at the diaphragm (b) or at the tentacle sheath (a). (c) Cyclostomes possess bundles of vestibular dilatators that extend from the distal bodywall to the diaphragm. Comparable to the duplicature bands, cyclostomes possess an attachment organ built by peritoneal strands. (d - h) Ctenostomes. (d) Paludicelloidean ctenostomes as *Paludicella *show homologs of phylactolaemate duplicature bands, parieto-vaginal bands, and prominent vestibular dilatators, which are subdivided into the proximal parieto-diaphragmatic muscles and the distal parieto-vestibular muscles. (e + f) Ctenostomes forming elongated peristomes/stolons usually lack parieto-vaginal bands and show a strong separation of parieto-diaphragmatic and distal parieto-vestibular musculature. Alcyonidioideans (g) possess parieto-vaginal bands, whereas hislopioideans (h) lack them. The vestibular dilatators consist of a single portion in hislopioideans. In Alcyonidioideans they are separated into parieto-diaphragmatic and distal vestibular musculature. (i) Cheilostomes possess parieto-vaginal bands and parieto-diaphragmatic musculature as found in ctenostomes. The aperture in cheilostomes is closed by the opercular occlusors - most likely modified distal vestibular muscles. Abbreviations: a - atrium, ao - attachment organ, db - duplicature bands, dvm - distal vestibular muscles, ms - membranous sac, oocl - operculum occlusors, op - operculum, pdm - parieto-diaphragmatic muscles, pvb - parieto-vaginal bands, sph - sphincter, v - vestibulum, vd - vestibular dilatators.

Apertural muscles are present in all three ectoproct subtaxa, the Phylactolaemata, the Stenolaemata (with the sole extant taxon Cyclostomata) and the Gymnolaemata, which include the Ctenostomata and Cheilostomata. A review of the existing literature shows that their terminology is utterly confusing and inconsistent. Common terms found for apertural muscles are for example "parieto-vaginal muscles" [26], "parieto-diaphragmatic muscles" [31], "parieto-atrial muscles" [32], "parieto-vestibular muscles" [33], "pyramidal muscles" [34] or "longitudinal parietal muscles" [35]. The latter term has been established according to the notion that apertural muscles are derived parietal muscles [28,33] and is also frequently used in more recent compendia on ectoprocts [35-37]. A comparison of these muscles is most easily performed among retracted zooids (Figure 7). In all ectoproct subtaxa, retracted zooids show a distalmost invaginated portion of the cystid wall, termed the vestibular wall, which is separated from the proximally adjoining tentacle sheath by a diaphragm. At the diaphragm a strong sphincter is present in all three subtaxa (Figure 7).

The Phylactolaemata, the suggested sistergroup of the remaining ectoprocts [38,39], have two different apertural muscle systems: the duplicature bands and the vestibular dilatators (Figure 7a, b). The duplicature bands are peritoneal bands containing longitudinal muscles fibres that emerge from the lateral bodywall and insert either directly at the diaphragm (as in the lophopodids: Figure 7b; *Lophopus*: [34,40]; *Lophopodella*: [41]) or at the tentacle sheath below the diaphragm in the remaining taxa (Figure 7a; *Cristatella*: pers. obs., *Fredericella*: [34], *Pectinatella*: [33,34,42]; *Plumatella*: [34,43]; *Stolella*: [44]). Vestibular dilatators consist of separate muscle fibres that traverse the coelom distally of the duplicature bands. In all phylactolaemates they are loosely arranged and run from the lateral bodywall towards the vestibular wall. They attach to the entire area of the vestibular wall, starting at the area of the diaphragm and projecting up to the distal parts of the vestibular wall.

Cyclostome ectoprocts show a peculiar coelomic condition. In contrast to all other ectoprocts, the peritoneal layer of the endocyst is detached from the epidermis to form a coelomic sac, the membranous sac, around the polypide (Figure 7c; [33,45]). However, topographically similar to the duplicature bands of phylactolaemates, cyclostomes possess an attachment organ. It consists of ligaments that attach the polypide and membranous sac to the skeletal walls of the zooid (Figure 7c; [45,46]). Supposedly homologous structures of the phylactolaemate vestibular dilatators are present as few muscle fibres ("musculi extensores vestibuli" *sensu *[47], "longitudinal ectodermal muscle" *sensu *[45]), that run from the distalmost bodywall (i.e., terminal membrane in cyclostomes) to the diaphgram (Figure 7c).

In principle, the Gymnolaemata (Cteno- and Cheilostomata) also possess two muscular systems. First, homologs of the duplicature bands, which are most commonly termed "parieto-vaginal bands", second, vestibular muscles that are always prominent in cteno- and cheilostomes.

Protoctenostome, i.e. benedeniporoidean [25], polypide morphology is only insufficiently known. 'Parieto-vaginal muscles' were mentioned for *Benedenipora catenata *[48], but their description and illustration are too incomplete for drawing any comparisons. The vestibular muscles in ctenostomes usually form two portions, a small proximal portion of muscular bundles that insert at the diaphragm, the parieto-diaphragmatic muscles, and a large distal portion that attaches to the vestibular wall. The latter muscles are often referred to as parieto-vaginal muscles. To avoid confusion with the parieto-vaginal muscle bands, we refer to them as "distal vestibular muscles". By comparison with the potential outgroups, the Phylactolaemata and Cyclostomata, the presence of these two sets of muscles can be regarded as the plesiomorphic condition. An erect and simple, uniserial colony morphology is regarded as ancestral among ctenostomes [5,22], a condition that among "carnosan" ctenostomes is most closely exhibited by the paludicelloideans. They possess parieto-vaginal bands and their vestibular muscles are present as parieto-diaphragmatic muscles that consist of few fibres closely adjoining the distal vestibular muscles (Figure 7d; *Paludicella*: [49-52]).

The 'carnosan' superfamilies Alcyonidioidea and Hislopioidea are both considered early offshoots within ctenostomes (Figure 1). Alcyonidioideans possess parieto-vaginal bands (*Alcyonidium*: [53-55]; *Elzerina*: depicted, but not labelled by [1]) and their vestibular muscles show a distinct separation into parieto-diaphragmatic and distal vestibular muscles (Figure 7g; e.g., Alcyonidium: [53]). Within the Hislopioidea the current study on *Hislopia malayensis *shows that parieto-vaginal bands are absent and only vestibular muscles are present, as reported by Annandale [52]. A similar arrangement of muscles is present in *H. corderoi *[56,57]. Only for *H. lacustris *a set of muscles resembling parieto-vaginal bands has been illustrated [58]. However, the investigated specimens were not well preserved and differences in the muscular system were stated to be absent among different species of *Hislopia *[52]. Accordingly, it is appropriate to assume general absence of parieto-vaginal bands for hislopiid ectoprocts. In contrast to the Alcyonidioidea, vestibular muscles of *Hislopia *show no separation into parieto-diaphragmatic and distal vestibular muscles, but instead extend over the entire length of the vestibular wall (Figure 7h).

Arachnidioidean ctenostomes display a wide range regarding the length of the peristome and are mainly characterized by cystid appendages which often anastomose among individual zooids of a colony. Details on their polypide morphology are mostly restricted to the genus *Nolella *[59,60], which always has an elongated peristome. So far, no parieto-vaginal bands have been recorded for this genus (Figure 7e).

The Victorelloidea, Walkerioidea, and Vesicularioidea are characterized by peristome-elongation/trophon or stolon formation [22,25] with often reduced parieto-vaginal bands. In the latter two clades, the distal parieto-vaginal muscles are spatially more displaced from the parieto-diaphragmatic muscles (Figure 7f; e.g., *Bowerbankia*: [26]) than in the victorelloideans (*Victorella*: [26]) or the arachnidioideans (e.g., *Nolella*: [59]). Within the Walkerioidea only *Hypophorella expansa*, a species considered to be basal within this clade [25], possesses distinct parieto-vaginal bands [53,61]. Indications for the latter are also present in *Farrella repens *[62], while all other walkerioideans have reduced them (*Aeverillia*: [63]; *Harmeriella*: [64]; *Walkeria*: [65,66]). Similarly, a single species among victorelloideans, *Sundanella sibogae*, possesses parieto-vaginal bands [67], while other genera have reduced them (*Pottsiella*: [68,69]; *Victorella*: [26,70]). From our current knowledge, all Vesicularioidea lack parieto-vaginal bands (*Bowerbankia*: [26,52,59,71,72]; *Buskia*: [73]; *Spathipora*: [74]; *Terebripora*: [75]; *Vesicularia*: [76]).

Parieto-vaginal bands are present in malacostegan ectoprocts (Table 1), which are commonly regarded as a paraphyletic to all remaining Cheilostomata [77]. Consequently, parieto-vaginal bands are probably part of the ancestral cheilostome bauplan and may have been present in the last common cheilostome ancestor (Figure 7i). Since they have also been recorded in species from almost all "higher" groupings of cheilostomes (Table 1 and references therein [31,59,78-92]), it seems reasonable to assume that they are present in most cheilostomes. Cheilostomes have retained the parieto-diaphragmatic muscles. Distally to these muscles, opercular occlusors are situated (Figure 7i). Based on their similar topology and insertion points, we consider the occlusors as a modification of the distal vestibular muscles. As a consequence, we reject the notion that apertural muscles, including the opercular occlusors, are phylogenetically derived from parietal muscles [28,33]. As shown above, apertural muscles are present in all three ectoproct subclades including the Phylactolaemata and Stenolaemata, which both lack parietal muscles.

**Table 1:** List of cheilostome genera where parieto-vaginal bands have been found

Cheilostomata					
	Suborder	Superfamily	Family	Genus	Reference
**"Anasca"**					

	Malacostegina	Membraniporoidea	Membraniporidae	*Membranipora*	[30,77,78]
			Electridae	*Electra*	[58,79,80]
	Flustrina	Flustroidea	Flustridae	*Flustra*	[81,82]
				*Carbasea*	[81,83-85]
		Calloporoidea	Tendridae	*Tendra*	[86]
		Buguloidea	Bugulidae	*Bugula*	[58]
		Microporoidea	Microporidae	*Andreella*	[87]
			Steginoporellidae	*Steginoporella*	[84]
**Ascophoran**					

	Hippothoomorpha	Hippothooidea	Hippothoidae	*Antarctothoa*	[88]
	Umbonulomorpha	Lepralielloidea	Umbonulidae	*Umbonula*	[83]
	Lepraliomorpha	Schizoporelloidea	Cryptosulidae	*Cryptosula*	[58,89]
			Eminooeciidae	*Eminooecia*	[90]
		Smittinoidea	Smittinidae	*Parasmittina*	[91]

Several authors have regarded the cheilostome ancestors to be *Arachnidium*-like [1,28], but as previously mentioned, soft body morphology is almost unknown for the genus. The most precise data are available for the genus *Nolella*, which forms elongated peristomes. Similar to *Nolella*, species with long peristomial tubes that belong to other superfamilies commonly lack the parieto-vaginal bands. Since parieto-vaginal bands were probably present in the ctenostome-like ancestor of cheilostomes, it would be of particular interest to study arachnidioidean species with short peristomes, such as *Arachnidium fibrosum*.

### Lophophoral and digestive tract musculature

The tentacle musculature consists of two longitudinal muscle bands in most ectoprocts investigated to date [33]. Within Phylactolaemata, *Fredericella *is an exception in having only one longitudinal muscle band [93]. In general, the tentacle musculature is smooth in phylactolaemates and cheilostomates, whereas striated myofibrils have been reported for ctenostomes and cyclostomes. However, only for phylactolaemates several species were analysed in detail by electron microscopy [33,93]. Accounts on cyclostome tentacle muscles rely on the classical study of Borg [47], whereas only a single species of both cteno- [94] and cheilostomes [95] were analysed on the ultrastructural level. We observed striation of the longitudinal tentacle musculature of *Hislopia malayensis*, which supports the previous notion that ctenostomes possess striated tentacle musculature.

As the center of the nervous system and source of feeding and sensory structures, the lophophoral base represents the most complex part of the polypide [33]. However, most of our current knowledge resides in its description of the cheilostome *Cryptosula pallasiana *[95]. Buccal dilatators are present in this species, but were also described for the cheilostome *Bugula simplex *[59], the ctenostome *Bowerbankia pustulosa *[96], *Crisia eburnea*, and other cyclostomes [47,97]. They are smooth in *H. malayensis*, *C. pallasiana*, and *B. simplex*, while they were reported striated for *B. pustulosa*. A second pair of dilatators inserting above the ganglion as in *H. malayensis *has not been reported for any other species. In *C. pallasiana*, so-called "basal transverse tentacle muscles" are present at the base of each pair of tentacles that probably act as antagonist to the tentacle musculature [95]. These muscles are absent in *H. malayensis*, but possibly the circular ring muscle at the lophophoral base of this species may be a homologous structure. The two muscle groups at the lophophoral base below each longitudinal tentacle muscle have to our knowledge not been reported for any other ectoproct so far. Functionally, they remain difficult to interpret, but they could act in lophophore movement and rotation. To clarify their function, more detailed observations on living specimens, e.g., by video microscopy, are required.

In several ctenostomes the vacuolated cells of the pharyngeal epithelium possess distinct striated muscle fibres on their lateral walls. For *Zoobotryon verticillatum *[98] and *Alcyonidium polyoum *[99] ultrastructural analysis revealed that the pharyngeal cells along with these fibres represent a prominent myoepithelium. Light microscopical observations on such fibres were also conducted for the ctenostomes *Alcyonidium hirsutum *[100], *Victorella pavida *[101], and *Bowerbankia pustulosa *[96]. This specialized pharynx acts as a strong sucking pump for particle capture and has also been observed in the cheilostomes *Bugula flabellata *[100], *B. neritina *[102], and *Cryptosula pallasiana *[103]. In the current study we found no muscular elements in the pharyngeal cells of *Hislopia malayensis*. The only musculature associated with the pharynx consists of striated ring muscles surrounding the pharyngeal epithelium. Pharyngeal ring musculature has been described by the above mentioned authors for the respective species and is, when mentioned, striated. This musculature is also present in *Flustrellidra hispida *[104] and *Zoobotryon verticillatum *[105]. Longitudinal muscles in the pharynx were only found in *B. pustulosa *[96]. Most phylactolaemate bryozoans possess striated ring musculature in the pharynx as well [40,106]; only in *Asajirella gelatinosa *longitudinal muscles have been observed [33]. Cyclostome pharyngeal musculature consists of striated ring musculature and few longitudinal muscle fibres [47]. In conclusion, striated ring musculature appears to be a common trait of the ectoproct pharynx, whereas longitudinal fibres only occur in a few of the species studied so far. However, drawing the border between the pharynx and adjoining esophagus is often difficult in many ectoprocts and mostly only discernable by the ciliation pattern [107]. Accordingly, some of the observed longitudinal pharyngeal musculature could actually belong to the esophagus, which possesses few longitudinal fibres in *H. malayensis *(this study), *F. hispida *[104], and *B. pustulosa *[96].

The differentiation of the cardiac portion of the stomach or proventriculus into a gizzard occurs in some cheilostomes and cyclostomes, but is more frequently found in ctenostomes [33,108]. In the latter, the gizzard was previously considered to have evolved only once [25], while other authors argue for its multiple independent origin [108]. Internally, it is lined by cuticular plates or teeth used for crushing ingested food particles. In hislopiids the proventriculus carries a smooth cuticular lining in all species except for *Echinella placoides*, where it contains several spirally arranged cuticular ridges [109]. Functionally, it was either interpreted to act as a mere storage organ or as crushing organ for ingested food particles [110]. In *H. malayensis *the inner walls of the proventriculus never form contact and food particles are very small that do not require grinding. In the proventriculus, there is a very active exchange of undigested whole particles: the distal sphincter closes; then a slow wave of tight constriction starts from the proximal end which then moves distally. It squeezes the contents and forces them to spurt back through the narrow constriction, between cuticular ridges, towards the proximal end. In addition, particles are pushed back and forth between the lower proventriculus and the upper caecum. This is achieved mostly by cilia in these two regions but also by spasmodic contractions of the caecum (Wood, personal observation). The purpose of all this movement is unclear. The effect may be to break apart any particles that cling together and not to grind any particles.

In *H. malayensis *the remaining stomach or caecum possesses mainly smooth circular muscle bands with two prominent longitudinal muscles at the proximal side and few longitudinal fibres between the circular muscles. A similar muscular system of the caecum has been described for *F. hispida *[104], *Z. verticillatum *[105], *B. pustulosa *[96], and the cheilostome *C. pallasiana *[103]. The circular muscle bands in these species are not adjoining, but keep a distance to each other. On the contrary, phylactolaemates solely possess a dense layer of circular, striated muscle fibres in the caecum [33]. The remaining digestive tract, i.e. the intestine and rectum, possess only smooth longitudinal musculature in *H. malayensis *(this study), *Z. verticillatum *[105], and *B. pustulosa *[96], while additional ring musculature was described for *F. hispida *[104]. In contrast, most phylactolaemates possess mainly densely packed smooth ring musculature in the intestine [40]. Among phylactolaemates, only *A. gelatinosa *shows few additional longitudinal muscle fibres in the intestine [33].

### Retractor muscles and the striation problem

The partitioning of the zooid into the cystid and a retractable polypide is a characteristic feature of all ectoprocts. Accordingly, polypide retractors are present in all clades, and usually constitute the most prominent somatic muscles. Their traverse within the coelom is usually simple and unidirectional from the proximal or lateral cystid wall to the lophophore base [111]. This condition is also present in *H. malayensis *and other species of *Hislopia *[52,56,57].

Retractor muscle fibres have been controversially described as either striated (e.g. [54,112]) or smooth (see [113]). Thereby, all ultrastructural studies found the fibres to be smooth [33]. In the current study on *Hislopia malayensis *we found the retractor muscle fibres to be mainly smooth, while the distal-most parts appeared 'striated'. A similar appearance of the retractor muscle fibres was recently observed in *Pottsiella erecta *[69]. Such striations were attributed as 'pseudostriations', resulting from contraction folds or helically coiled fibrils [73,96,113]. However, other ctenostomes also show striations in expanded zooids with relaxed retractors (Schwaha: unpublished observations). Consequently, it remains difficult to fully interpret these striation patterns, which inevitably requires ultrastructural analysis.

### Myogenesis during the budding process

The temporal appearance of the retractor, apertural and parietal muscles, as well as the polypide anlage during budding has previously been analysed in several ctenostomes and has been used for phylogenetic inferences. The earliest analyses tried to reconstruct the suborders 'Carnosa' and 'Stolonifera' as monophyletic taxa [23,24], which was later rejected [22,25]. The latter author, Jebram [22], analysed myogenesis during the budding process of a hislopioid, *Hislopia corderoi*. Accordingly, parietal muscles are the first to appear during budding in *H. corderoi*, whereas the polypide anlage is second, followed by the retractor muscles and the apertural muscles, respectively - a succession also found in specimens of the closely related superfamilies Alyconidiodea and Arachnidioidea [22]. The results of this study on *H. malayensis *show that the parietal muscles are last to appear during budding, which is not in accordance with the observations on *H. corderoi*. However, it has to be considered that the observations by Jebram [22] were conducted on old preserved material and that the specimens were mainly analysed as whole mounts. With these methods, the delicate first anlagen of the retractor and apertural muscles are rather difficult to distinguish. Considerable variation in the asexual muscle succession occurs among numerous other ctenostome species [22] and might also be present among species of *Hislopia*. As promising these muscle successions might be for ctenostome phylogeny, their value should perhaps not be overestimated until analysed with modern methods.

### The serotonergic nervous system

The serotonergic nervous system in Ectoprocta was previously analysed in different kinds of planktotrophic and lecitotrophic larvae [14-16,114]. During ectoproct metamorphosis, larval organ systems undergo histolysis and adult organs, including the nervous system, are formed anew ("catastrophic metamorphosis") [16,115]. Due to the significant morphological differences, the individual components of the larval nervous systems are most likely lost during metamorphosis and are thus not considered ontogenetically homologous sensu Haszprunar [116]. So far, the adult serotonergic nervous system has only been investigated in the lepraliomorph cheilostome *Triphyllozoon mucronatum *[16]. The latter shows a similar condition to *Hislopia malayensis*. The highest concentration of serotonin in both species is located in the circumpharyngeal nerve ring or "cerebral ganglion". Additional serotonergic perikarya are present at the lophophoral base and are connected to the ganglion by fine neurites. *Hislopia malayensis *possesses additional serotonergic neurites, which run from these perikarya into the tentacles. Such serotonergic neurites have not been found in *T. mucronatum*. These results currently support a common serotonergic nervous system in gymnolaemates, i.e. cteno- and cheilostomes, but for estimating its value for phylogenetic considerations more species need to be analysed.

## Conclusions and Outlook

This study presents the first data on the myoanatomy and serotonergic nervous system of an adult ctenostome ectoproct, *Hislopia malayensis*. Comparative analysis revealed that the Phylactolaemata show several morphological differences to the remaining ectoprocts by, e.g., being the only ectoproct taxon with a distinct bodywall musculature. Only few synapomorphies for all ectoproct taxa are identifiable (cf. [93]). As an example, our study demonstrates that the apertural muscles are highly similar among the major ectoproct subclades and consist of two principal sets of muscles, the parieto-vaginal bands and the vestibular muscles. These have been modified among the different ectoproct taxa according to the different morphology of the aperture. Their phylogenetic derivation from parietal muscles seems unlikely. Concerning the apertural muscles in ctenostomes, two main evolutionary trends are apparent: 1. Formation of dense encrusting colonies with almost box-shaped zooids and the aperture and its associated musculature shifted towards the frontal side (Figure 7g, h). 2. Peristome elongation or even stolon formation, usually accompanied by loss of parieto-vaginal bands and with distal parieto-vaginal musculature being more distantly situated from the parieto-diaphragmatic musculature (Figure 7e, f). Parieto-vaginal bands were most likely present in the cheilostome ancestor and appear to be present in most extant Cheilostomata.

Concerning the musculature of the digestive tract, there are clear differences between the Phylactolaemata and the Gymnolaemata. Most species of the former possess tightly packed, mostly cross-striated ring musculature, whereas the digestive tract musculature of the Gymnolaemata contains longitudinal muscles that are more loosely arranged. Among the phylactolaemates, only *Asajirella gelatinosa *possesses few longitudinal muscles in the wall of the digestive tract. This species belongs to the family Lophopodidae, which is currently regarded as the most basal family within Phylactolaemata [117,118]. Accordingly, we assume longitudinal and ring musculature in the wall of the digestive tract a basal ectoproct feature. Similarly, two longitudinal muscle bands in the tentacles of the lophophore seem to constitute a basal ectoproct feature. The lophophoral base connecting the tentacles shows a complex set of several muscle groups, but due to the lack of comparative data, conclusions on the plesiomorphic state for Ectoprocta are currently not possible.

Cyclostome ectoprocts possess annular ring muscles in their membranous sac that might have evolved from the ring musculature of the phylactolaemate bodywall [45]. However, additional investigations using state-of-the-art technology are needed to further address this issue, since most available data rely on the classical work by Borg [47].

## Competing interests

The authors declare that they have no competing interests.

## Authors' contributions

TS conducted all practical work and drafted the manuscript. TW coordinated research in Thailand, collected and identified the animals and contributed to the manuscript. AW provided immunocytochemistry and confocal microscopy facilities and contributed significantly to the writing of the manuscript. All authors read and approved the final version of the manuscript.
